# Accuracy of 3D-planned patient specific instrumentation in high tibial open wedge valgisation osteotomy

**DOI:** 10.1186/s40634-020-00224-y

**Published:** 2020-02-27

**Authors:** Sandro F. Fucentese, Patrick Meier, Lukas Jud, Gian-Luca Köchli, Alexander Aichmair, Lazaros Vlachopoulos, Philipp Fürnstahl

**Affiliations:** 1 Department of Orthopedics, Balgrist University Hospital, University of Zurich, Forchstrasse 340, 8008 Zürich, Switzerland; 2 Computer Assisted Research and Development Group (CARD), Balgrist University Hospital, University of Zurich, Zurich, Switzerland

**Keywords:** HTO, Computer-assisted planning, Patient-specific instrumentation, Medial compartment osteoarthritis of knee, High tibial open wedge osteotomy, Tibial slope

## Abstract

**Purpose:**

High tibial osteotomy (HTO) is an effective treatment option in early osteoarthritis. However, preoperative planning and surgical execution can be challenging. Computer assisted three-dimensional (3D) planning and patient-specific instruments (PSI) might be helpful tools in achieving successful outcomes. Goal of this study was to assess the accuracy of HTO using PSI.

**Methods:**

All medial open wedge PSI-HTO between 2014 and 2016 were reviewed. Using pre- and postoperative radiographs, hip-knee-ankle angle (HKA) and posterior tibial slope (PTS) were determined two-dimensionally (2D) to calculate 2D accuracy. Using postoperative CT-data, 3D surface models of the tibias were reconstructed and superimposed with the planning to calculate 3D accuracy.

**Results:**

Twenty-three patients could be included. A mean correction of HKA of 9.7° ± 2.6° was planned. Postoperative assessment of HKA correction showed a mean correction of 8.9° ± 3.2°, resulting in a 2D accuracy for HKA correction of 0.8° ± 1.5°. The postoperative PTS changed by 1.7° ± 2.2°. 3D accuracy showed average 3D rotational differences of − 0.1° ± 2.3° in coronal plane, − 0.2° ± 2.3° in transversal plane, and 1.3° ± 2.1° in sagittal plane, whereby 3D translational differences were calculated as 0.1 mm ± 1.3 mm in coronal plane, − 0.1 ± 0.6 mm in transversal plane, and − 0.1 ± 0.6 mm in sagittal plane.

**Conclusion:**

The use of PSI in HTO results in accurate correction of mechanical leg axis. In contrast to the known problem of unintended PTS changes in conventional HTO, just slight changes of PTS could be observed using PSI. The use of PSI in HTO might be preferable to obtain desired correction of HKA and to maintain PTS.

## Purpose

Development of osteoarthritis of the knee is often associated with a varus malalignment of the mechanical leg axis, resulting in an unbalanced load distribution in the joint. In younger patients, surgical treatments, such as high tibial osteotomy (HTO), are preferred over total knee arthroplasty [19, 23]. HTO can be also considered as the preferred surgical treatment over unicompartmental knee arthroplasty in patients with high activity requirements due to better postoperative range of motion [2].

One of the leading principles in HTO is to perform axis correction as precisely as possible because under- or over-correction is known to be the main reason for clinical failure [1]. Another challenge in HTO is the maintenance of the posterior tibial slope (PTS), wherefore a meta-analysis by Nha et al. [18] showed an increase of PTS in medial open wedge HTO of 2.0°, possibly caused by incomplete posterior corticotomy or due to a too anterior position of the fixation plate. However, unintended slope changes can result in anteroposterior knee instability and increased stress on the cruciate ligaments [10, 28, 30].

Planning of HTO in the conventional way is based on standing radiographs [20]. However, the surgical execution can be challenging without surgical navigation. It has already been shown that the use of patient specific instruments (PSI) improves precision of the reduction task in osteotomies in different orthopedic regions [14, 26, 29, 32]. Regarding HTO, Chaouche et al. [4] presented promising results using PSI in a study population of a hundred patients, however without three-dimensional (3D) accuracy assessment. Beside the high accuracy in PSI navigated HTO, also shorter operating times, a shortening of the learning curve, as well as a reduction of the used fluoroscopy time could be demonstrated by Jacquet et al. [12] and therefore further encouraging the use of PSI in HTO.

Goal of the present study was to assess the accuracy of PSI navigated HTO two-dimensionally (2D) by comparing the postoperative hip-knee-ankle angle (HKA) with the planned correction and the maintenance of the PTS, as well as in 3D by superimposition of the postoperative tibial bone models with the preoperative planning.

## Material and methods

The local ethical committee approved this study (Zurich Cantonal Ethics Commission, PB_2017–00075) and all patients gave their informed consent for their participation in and the publication of this study.

### Patient selection

All patients who underwent medial open wedge HTO using PSI from March 2014 until August 2016 were included. Indication for HTO was set in patients with medial varus osteoarthritis Grade 1–3 according to the Kellgren and Lawrence classification [15], with an age under 60 years and with failed conservative treatment. Cases performed without PSI or in which the surgeon intraoperatively intentionally modified the preoperative plan, were excluded. Patients who required an additionally correction of the PTS or patients with an intra-articular or femoral deformity were excluded likewise.

### Preoperative planning

For preoperative planning standard weight bearing radiographs of the knee, standing long leg radiographs, and computed tomography (CT) scan of the lower extremities were acquired. A CT protocol was developed in which the regions of interest (i.e. proximal and distal femur, knee center with medial and lateral eminences, ankle joint center with distal tibia, fibula and talus) were acquired, while reducing radiation exposure to the patient by skipping irrelevant mid-shaft regions. With the hereby-collected CT data, image segmentation was performed to reconstruct 3D triangular surface models as previously described in [3, 9, 27]. CT data was used due to the high-resolution images that allow accurate segmentation of the bones and therefore enable a detailed preoperative planning for the PSI.

**Fig. 1:**
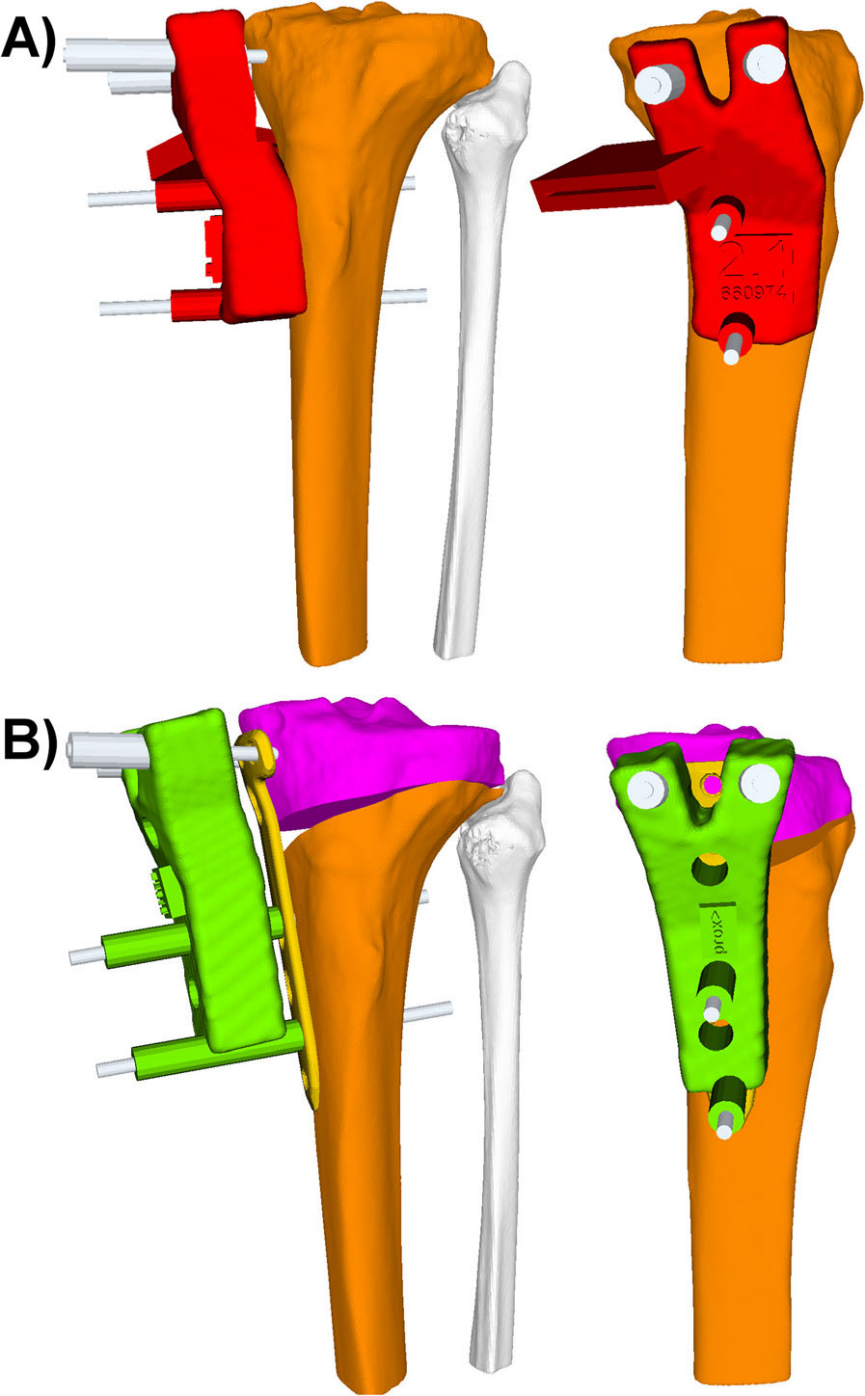
**a** In red the cutting-guide with the slot for the saw, in orange tibia before osteotomy. **b** In green post-reduction guide with pre-defined drill holes, in purple the proximal part and in orange the distal part of the osteotomized tibia. In gold the Tomofix Medial High Tibial Plate

Afterwards the models were imported into the preoperative planning software CASPA (Balgrist CARD AG, Zurich, Switzerland). The osteotomy plane was defined according to the TomoFix Medial High Tibial Plate surgical technique by DePuy Synthes [5]. To allow simulation of the HTO a hinge in the lateral tibial head was determined, at least 10 mm below the tibia articular surface, pointing in normal direction to the plane of correction. The hinge was placed on the osteotomy plane 3 mm medial to the lateral cortical surface of the tibia. While some authors propose to place an additional k-wire to prevent lateral hinge fractures [6, 11], in the here presented PSI system, the cutting depth is given through the integrated cutting slit on the PSI and wherefore in this guide design no additional k-wire was placed. Mechanical leg axis correction was simulated using a previously described computer algorithm [8] (capable of calculating the optimal slope-maintaining correction plane and the postoperative HKA in 3D). In most cases, the desired correction was planned by the use of the Fujisawa point, located on the proximal tibial joint line, 62.5% lateral from the medial tibial plateau edge [7]. However, in all cases the final planning of the correction was assessed and if needed adjusted by the treating surgeon, under consideration of the measured mechanical leg axis in the standing long leg radiographs (i.e. in weight-bearing condition) and under consideration of the complaints of the patient. In the last step of the preoperative planning, the Tomofix Medial High Tibial Plate (Depuy-Synthes Oberdorf, Switzerland) and the implant screws were positioned in 3D. PSI and 3D printouts of the native preoperative and simulative postoperative bones were manufactured using a selective laser sinter (Formiga P110, EOS, Germany) through Medacta (Medacta SA, Castel San Pietro, Switzerland) (Fig. 1). Biocompatible polyamid P2200 was used as the raw material. Before surgery, PSI and bone models were sterilized using conventional steam pressure sterilization in our institution.

### Surgical procedure

All surgical procedures were conducted or supervised by the senior surgeon (FS). Patients were placed in a supine position using general or spinal anesthesia. A tourniquet was applied and an anteromedial approach to the tibial head was performed. The bone was relieved of the soft tissue to identify prominent bony landmarks, which have been integrated in the undersurface of the PSI for proper positioning. Next, the basic guide, serving as a registration tool between the preoperative planning and the intraoperative situation, was positioned and reference pins for the orientation of the following guides were inserted. Screw positions of the Tomofix Medial High Tibial Plate (Depuy-Synthes Oberdorf, Switzerland) were pre-drilled using the integrated drill sleeves. Afterwards, the osteotomy guide was placed and the osteotomy was performed in a PSI-navigated fashion by predefined osteotomy-plane position and orientation, as well as cutting depth. Using the reduction guide, predefined reduction was performed and the Tomofix Medial High Tibial Plate could be placed over the pre-drilled screw holes, subsequently the screws could be successively inserted. Fluoroscopy was used to confirm osteotomy and positioning of the implants. Skin was closed over a drain.

### Aftercare

On the second postoperative day, plain radiographs of the knee were acquired. According to a standard aftercare protocol, patients were instructed to partially weight bare with 15 kg from the first day after the procedure for a period of 6 weeks. Follow-up was scheduled at 6 and 12 weeks after the procedure with standing long leg radiographs. CT scans to confirm bony consolidation were obtained at least 12 weeks postoperatively.

### Radiological outcome and accuracy assessment

Two independent readers performed radiological outcome assessment (MP, KG). 2D measurements were obtained using preoperative planning software (mediCAD®, module osteotomy; Hectec GmbH, Germany). Therefore, the mechanical leg axis was measured as HKA in standing long leg radiographs using the center of the femoral head, the knee and the ankle. The PTS was measured as the angle between the anatomical axis of the proximal third of the tibia and an average line between the medial and lateral tibia plateau. Pre- and postoperative HKA and PTS values were determined. 2D accuracy for HKA correction was calculated as the difference between planned HKA and postoperative HKA. 2D accuracy for PTS was calculated as the difference of pre- and postoperative PTS, accordingly as the maintenance of PTS. 3D measurements were conducted with the CASPA software as follows: 3D reconstructed proximal tibia models of the preoperative planning and the postoperative CT were superimposed to calculate deviation in 3D according to a method described by Schenk et al. [22]. Accordingly, 3D rotational accuracy was calculated as the difference between planned and postoperative proximal tibia models with respect to the coronal, transversal and sagittal planes. The 3D translation accuracy was quantified using the same planes.

### Statistical evaluation

Interrater reliability was analyzed using intraclass correlation coefficients (ICC). Intraclass correlation coefficients were interpreted according to Landis and Koch [16]. 2D and 3D precision was evaluated by comparing pre- and post-operative values, expressed by average and standard deviation. In these evaluations, the measurements of both readers were included. With regard to angular measurements, varus deformities were described as negative values, whereas valgus deformities were described as positive values. Pre- to postoperative changes in HKA were listed as the absolute values. Statistical evaluation was carried out with the software R (version 1.1.463; R foundation, Vienna, Austria).

## Results

The study population consisted of a total of 23 subjects (M: 16, F: 7) with an average age at time of surgery of 45.2 ± 7.6 (range: 25–58) years. The average BMI was 30.8 ± 6.3 (range: 21.5–46.3) kg/m^2^. Complications occurred in three patients. In one patient a surgical site infection occurred with *Staphylococcus aureus*, treated by surgical debridement and antibiotics with following uneventful healing. In two patients impairment of wound healing was observed without the need of an additional surgical therapy.

**Fig. 2:**
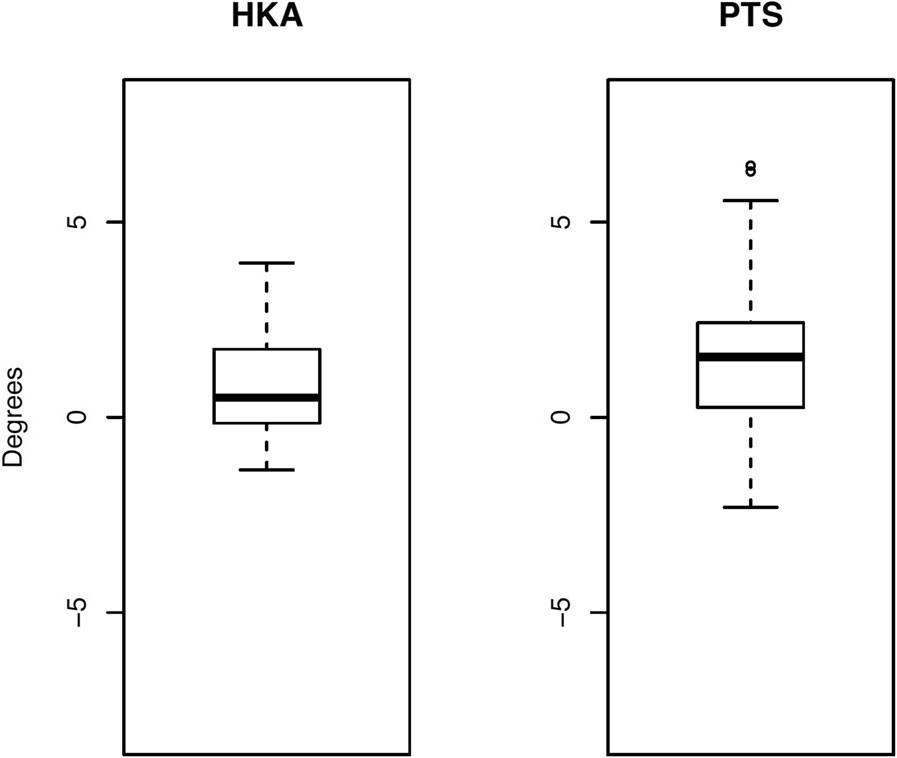
Statistical evaluation of the 2D accuracy of the mechanical leg axis (HKA) correction, measured as the mean difference of HKA between planned and achieved correction, and the average accuracy of maintaining posterior tibial slope (PTS). For graphical visualization, boxplots were used, with the ends of the whiskers indicating 1.5 times the interquartile range (IQR) between the lower and upper quartiles, and outliers denoted with a circle

Assessment of the preoperative 2D measurements showed a mean HKA of − 7.1° ± 3.0° and PTS as 6.4 ± 3.0°. Postoperatively, the mean HKA changed to 1.7° ± 2.5° and the PTS to 8.1° ± 3.5°. On average, a HKA correction of 8.9° ± 3.2° was performed, whereby a mean correction of 9.7° ± 2.6° was planned. The accuracy of the HKA correction was 0.8° ± 1.5° (Fig. 2). In all but six patients deviation from planned to achieved correction of HKA was less than 2°. The average accuracy of maintaining PTS was 1.7° ± 2.2° (Fig. 2) whereby in all but nine patients slope changes were < 2°. In the first twelve cases the average accuracy of maintaining PTS was 2.4° ± 2.2°, in the following eleven cases the average accuracy of maintaining PTS was 0.9° ± 1.8°.

**Fig. 3:**
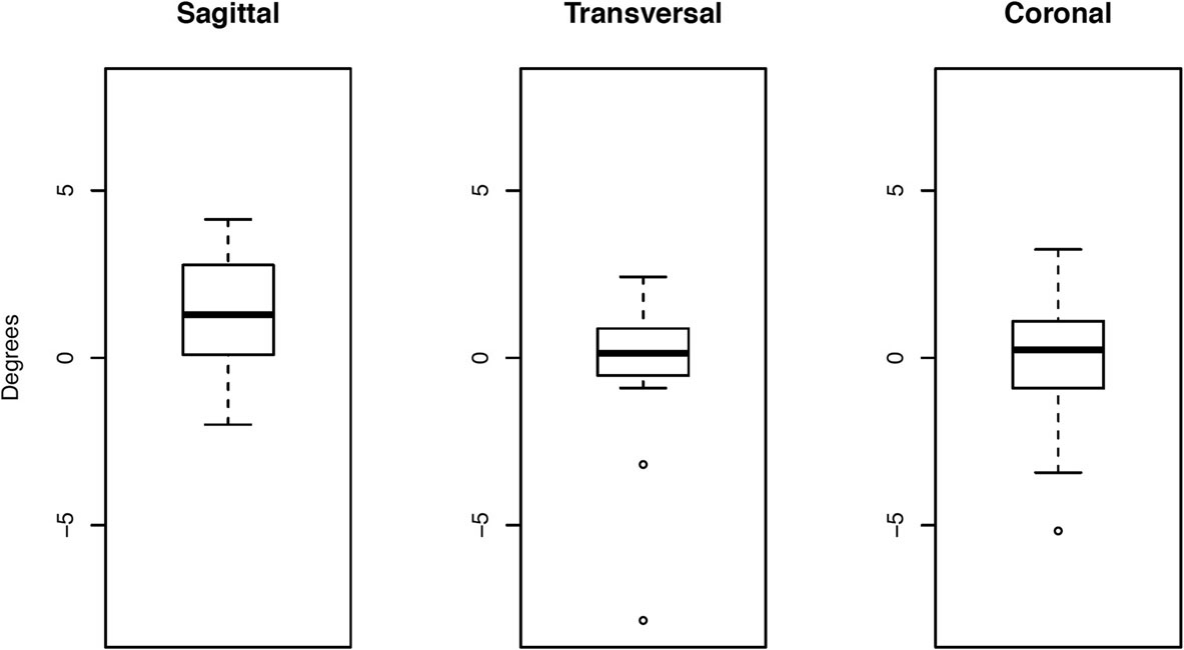
Statistical evaluation of the 3D accuracy of average rotational differences between preoperative planning and surgical execution in all the three plains. For graphical visualization, boxplots were used, with the ends of the whiskers indicating 1.5 times the interquartile range (IQR) between the lower and upper quartiles, and outliers denoted with a circle

3D accuracy assessment showed average 3D rotational differences between preoperative planning and surgical execution of − 0.1° ± 2.3° in the coronal plane, − 0.2° ± 2.3° in the transversal plane, and 1.3° ± 2.1° in the sagittal plane (Fig. 3). Assessment of the average 3D translational differences showed values of 0.1 mm ± 1.3 mm in the coronal plane, − 0.1 mm ± 0.6 mm in the transversal plane, and − 0.1 mm ± 0.6 mm in the sagittal plane.

The ICC between two readers for measuring 3D rotational accuracy showed “moderate” agreement in the coronal plane [0.52 (95%CI: 0.15–0.76)], also “moderate” agreement in the transversal plane [0.55 (95% CI: 0.17–0.78)], and “fair” agreement in the sagittal plane [0.21 (95% CI: − 0.21 – 0.57)]. ICC for measuring 3D translational accuracy showed “slight” agreement in the coronal plane [0.035 (95% CI: − 0.37 – 0.43)], also “slight” agreement in the transversal plane [0.086 (95% CI: − 0.33 – 0.47)], and “poor” agreement in the sagittal plane [− 0.076 (95% CI: − 0.47 – 0.34)]. The ICC between two readers for 2D parameters showed “almost perfect” agreement for preoperative [0.99 (95% CI: 0.98–1)] and postoperative [0.98 (95% CI: 0.96–0.99)] HKA. Likewise preoperative [0.92 (95% CI: 0.82–0.97)] and postoperative [0.89 (95% CI: 0.76–0.95)] PTS showed “almost perfect” agreement.

## Discussion

The most important finding of the here present study is, that high accuracy in navigating HTO with the used PSI system could be confirmed. Concerning the fact that under- or over-correction is the main reason for clinical failure in HTO [1], leading principle in such procedures should be to achieve the highest accuracy possible. A systematic review by Van den Bempt M et al. [24] showed eight out of fourteen cohorts treated by conventional HTO with less than 75% of patients being postoperatively in an acceptable range of accuracy. Therefore, new techniques are desirable to perform the surgery more precisely according to the preoperative planning. In the current literature, five studies [4, 17, 21, 25, 31] exist, investigating the accuracy of PSI for navigation of HTO in a patient population. The latest study of Chaouche et al. [4] investigated a hundred patients that underwent HTO using PSI and showed an accuracy from planned to achieved HKA correction of 1° ± 0.95°. Yang et al. [31] investigated ten patients that underwent HTO using PSI whereby the correction was planned in all patients on the Fujisawa point, 62.5% lateral from the medial tibial plateau edge. Achieved correction was in mean 60.2% ± 2.78% lateral from the medial tibial plateau edge. Mean PTS changed from preoperative 9.9° to 10.1° ± 0.4°. Another study by Munier et al. [17] evaluated accuracy also in ten patients. They reported optimal correction in a safe and reliable manner. In none of their patients difference of planned and achieved HKA was > 2°. In one patient deviation of postoperative PTS correction was > 2° than planned. Except this study, Munier’s study was the only one also analyzing the accuracy of the correction in 3D. They reported errors of less than 2° in the coronal and sagittal planes in all but one patient. Perez-Mananez et al. [21] reported accuracy of PSI for HTO navigation of 2°, evaluated on coronal radiographs. The PTS however was not assessed in their study. Another study of Victor et al. [25] investigated the use of PSI in osteotomies around the knee. Their planning did not rely on anatomical measurements, instead they used a mirror-model of the contralateral bone as a reconstruction template. They reported an accuracy of 1° in the coronal plane and of 2° in the sagittal plane.

By using the here presented PSI system for the navigation of HTO, a mean HKA-correction accuracy of 0.8° ± 1.5° could be achieved. In two out of twenty-three patients, the deviation from planned correction was greater than 3°. One reason for the imprecise execution in these cases could be inaccurate placement of the PSI and a learning curve. The PSI used in this study relied solely on prominent bony landmarks, needed for correct placement. However, in a computer simulation study, it could be shown that additional stabilizing arms on the PSI are able to further lever up the accuracy of PSI navigated HTO [13]. Therefor it can be assumed that newer guide design generations would results in even better accuracy as already achieved with the here presented guide design.

This study also evaluated how precise the preoperative PTS could be maintained after correction. A mean PTS change of 1.7° ± 2.2° was measured, which is lower compared to 2.0° reported previously in the meta-analysis by Nha KW et al. [18]. While the average accuracy of maintaining PTS was 2.4° ± 2.2° in the first twelve case, in the following eleven cases the average accuracy of maintaining PTS was 0.9° ± 1.8°. This is probably a result of to the learning curve.

Regarding the ICC for 3D analysis between the two readers with just “poor” to “moderate” agreement it has to be noted, that superimposition of 3D reconstructed models is a complex procedure with dependence on the investigator. Several steps, aligning the 3D reconstructed models are needed, wherefore just slight deviations between readers add up, resulting in these undesirably ICCs. To date superimposition of 3D reconstructed models is not a standardized procedure yet. Referring to the more simple 2D measurements the ICC showed to be “almost perfect” for all measurements.

A limitation of the here presented study is the limited sample size. Nevertheless it has to be noted that the patient population is equal or even bigger than other studies investigating accuracy of PSI in HTO [17, 21, 25, 31]. Secondly, patients in whom additional correction of PTS was aimed were excluded. Reason for this exclusion criterion was that in particular the accuracy of maintaining the preoperative slope was one objective of this study. Third, it has to be mentioned, that performing HTO using PSI needs an equivalent skin incision to place the implants, and minimal invasive PSI procedures are not yet available. Nevertheless in order to achieve highest accuracy possible, it should justify this circumstance so far. Lastly, it has to be noted that a control group with conventional HTO would be valuable to confirm the advantages of the use of the here presented PSI system. However, we wanted to understand the potential but also the risks of this new technology. So we decided that it makes more sense to include as much cases as possible in the PSI group to compare these results with the huge literature of conventional HTO.

## Conclusion

High accuracy for HKA correction using the here presented PSI system could be confirmed, while also preserving the PTS. However, for maintaining PTS, a learning curve could be observed. An additional prospective study comparing conventional HTO with HTO using the here presented PSI system would be needed to confirm superiority of this system.

## Data Availability

Anonymized source data can be obtained from the corresponding author on reasonable request.

## Abbreviations

2D: Two-dimensional
3D: Three-dimensional
CT: Computed tomography
HKA: Hip-knee-ankle angle
HTO: High tibial osteotomy
ICC: Intraclass correlation coefficients
PSI: Patient-specific instruments
PTS: Posterior tibial slope
